# Root Morphological Traits and Spatial Distribution under Different Nitrogen Treatments and Their Relationship with Grain Yield in Super Hybrid Rice

**DOI:** 10.1038/s41598-017-18576-4

**Published:** 2018-01-09

**Authors:** Ke Liu, Aibin He, Chang Ye, Shaowen Liu, Jian Lu, Mengtao Gao, Youzhong Fan, Bilin Lu, Xiaohai Tian, Yunbo Zhang

**Affiliations:** 1 Hubei Collaborative Innovation Center for Grain Industry, Yangtze University, Jingzhou, Hubei 434025 China; 2 Agriculture College, Yangtze University, Jingzhou, Hubei 434025 China

## Abstract

Succeeding in breeding super hybrid rice has been considered as a great progress in rice production in China. This on-farm study was conducted with Minirhizotron techniques to identify dynamic root morphological traits and distribution (0–30 cm) under different nitrogen treatments. Five elite super hybrid rice cultivars, Liangyoupeijiu (LYPJ), Yliangyou 1(YLY1), Yliangyou 2(YLY2), Yliangyou 900(YLY900) and Super 1000(S1000), were grown at four N levels: 0 kg ha^−1^ (N1), 210 kg ha^−1^ (N2), 300 kg ha^−1^ (N3) and 390 kg ha^−1^ (N4) in 2015 and 2016. Results showed these cultivars had greater root traits and higher grain yield under N3. Total root number (TRN) and total root length (TRL) of these cultivars reached maximum at 55 days after transplanting (DAT). The new released cultivars YLY900 and S1000 were featured with an improved root system among these cultivars. The percentage of root number on 10–20 cm soil was over 50% compared with other soil layer. A significant positive correlation was found between grain yield and both TRN and TRL at 10–20 cm soil layer (*P* < 0.01). Given this situation, the grain yield of super rice cultivars could be further improved by increasing the proportion of roots at 10–20 cm soil layer under suitable nitrogen management.

## Introduction

To meet food demand of growing population in the 21st century, a super-rice breeding program aimed at increasing productivity started in China since 1996^1^. Afterwards, China’s super hybrid rice cultivars adopting a combination of the ideotype approach and inter-subspecific heterosis have been released^2,3^. By 2017, 130 rice cultivars have met the criteria of super rice cultivars, which are released by Ministry of Agriculture of China (http://www.moa.gov.cn/zwllm/tzgg/tz/index_5.htm). The super rice breeding program was divided into four phases, and the representative cultivar of Phase I, named Liangyoupeijiu, Yliangyou-1 (Phase II), Yliangyou-2(Phase III) and Yliangyou-900(Phase IV) have achieved the yield targets respectively, under optimal cultivation practices^1^. Currently, great efforts have been made to achieve new yield targets with another elite super hybrid rice cultivar, Super-1000(Phase V). These super hybrid rice cultivars have an average of 10% yield advantage than ordinary hybrid cultivars^4–6^. These elite cultivars with high yield potential have made extraordinary contribution to food production and provided an excellent opportunity to identify novel features associated with the increased yield potential. So far, several morphological and physiological traits contributing to the higher yield in super hybrid rice have been documented on its aboveground parts, such as larger sink size^7^, higher leaf area index^8^, slower leaf senescence^9^, higher photosynthetic rate and stronger lodging resistance^10^, higher biomass accumulation before heading^11^, and higher translocation efficiency to grains during the grain filling period^9,12^.

In recent years, although the mean grain yield of super hybrid rice cultivars have increased, many studies showed that the difference in the harvest index between super hybrid rice cultivars and ordinary hybrid rice cultivars is not significant^13^. As harvest index has already reached a very high level(>0.5), the key to breakthrough of yield potential in future breeding of super hybrid rice should maintain the current harvest index and increase biomass production. Compared with inbred cultivars, super hybrid rice cultivars display better morphological features, such as higher biomass production, lower panicle position, and greater panicle size^14^. It is widely accepted that the high yield potential of super hybrid rice requires a large amount of N fertilizer input. However, farmers are likely to overuse the fertilizer application as they always apply a higher amount of N fertilizer in order to obtain grain yield which is far more than the plants’ requirement. As a result of this, global N consumption increased approximately 8-fold from 1961 to 2013, of which China accounted for approximately 8% in 1961 and 35% in 2013^15^. Therefore, studies on the optimum nitrogen rates for super hybrid rice have become a hot topic over the last few years. It has been reported that super hybrid rice had higher grain yield (11.42 t ha^−1^) under 245.9 kg ha^−1^ N rate, while the economic benefit was highest at 213.5 kg ha^−1^ N rate^16^. Other research pointed out that super hybrid rice cultivars, under the treatments with 300 kg ha^−1^ N rate, reached a good balance in both higher grain yield and higher nitrogen use efficiency^17^. However, these results are controversial since very high N application rates were used in their study.

On the other hand, root development is remarkably fluctuant to variations in nitrogen supply and its distribution in the soil^18^. Despite the fact that root system is involved in the absorption of nutrients and water, the synthesis of plant hormones and also the anchorage of the plants^19,20^, limited studies have been conducted on root morphological traits and spatial distribution of super hybrid rice under different nitrogen treatments, such as total root number (TRN), total root length (TRL), total root surface area (TRSA) and total root volume (TRV)^21,22^. This is partly due to technical difficulty in root determination^23^. Previous research observed that root numbers showed positive response to nitrogen concentration, while root length and diameter showed negative response^24^. Compared with high nitrogen treatment, rice roots under low nitrogen application had higher total surface area, root number and longer root length throughout the growth season^25^. Graeme *et al*. reported that changes in root system architecture and associated capture of water were important for increase in historical yield trends in maize^26^, but limited information is available on coordinating growth between root and shoot, and the changes that occurred to the root characteristics during different growth and development stages of the super hybrid rice. Given the close functional interdependence between roots and shoots, studying the root systems is likely to provide excellent opportunity to figure out the key factors that contribute to the yield potential of super hybrid rice cultivars. Therefore, the objectives of this study were (1) to compare root characteristics among super hybrid rice cultivars under different nitrogen treatments, (2) to identify morphological traits of roots which are responsible for the differences in grain yield among super hybrid rice cultivars under different nitrogen treatments, and (3) to find the possibility to further increase yield potential by regulating rice root growth with suitable nitrogen managements.

## Results

### Temperature during rice-growing seasons

**Figure 1:**
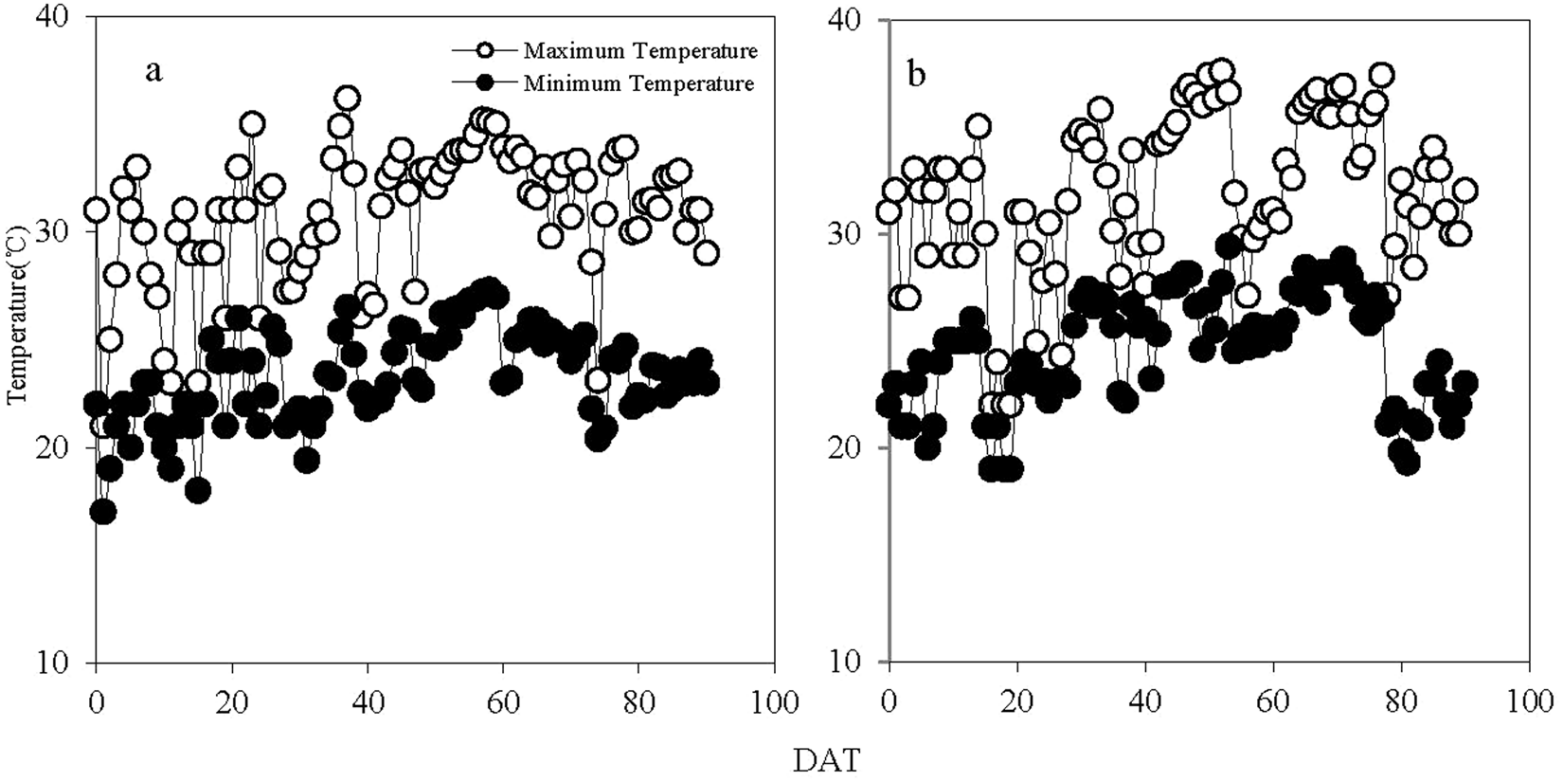
Daily maximum and minimum temperatures during rice-growing season in Jingzhou, Hubei Province, China, in 2015 (**a**) and in 2016 (**b**). DAT: days after transplanting.

Daily maximum and minimum temperature tended to be increasing with days passing after transplanting in 2015 and 2016, despite some fluctuations (Fig. 1a and b). The differences in mean daily temperature between 2015 and 2016 during the vegetative phase were relatively small (0.8–1.4 °C), but the differences were obviously significant during the reproductive phase, especially the anthesis. Mean daily maximum and minimum temperatures during the anthesis were 35.8 °C and 27.5 °C in 2016, respectively. Both were about 4 °C higher than those in 2015.

### Grain yield and yield components of the five super hybrid rice cultivars

Grain yield of all the cultivars varied significantly across the two years (Tables 1 and 2). The rates of N applied had a significant effects on grain yield (*P* < 0.01) in 2016 and the interaction between N and cultivar on grain yield was also significant (*P* < 0.05) in 2015. There was a positive correlation between N rate and grain yield for all cultivars within a limited N rate, but these cultivars showed a different pattern in response to N rate. LYPJ, YLY1 and YLY2 reached the highest grain yield under N2 treatment at 10.2 t ha^−1^, 11.1 t ha^−1^ and 10.3 t ha^−1^, respectively in 2015, while YLY900 reached the highest grain yield under N3, at 11.8 t ha^−1^ and 11.0 t ha^−1^ in the two years.

**Table 1:** Grain yield and yield components of the five super hybrid rice cultivars under the four N treatments in 2015.

**N rate**	**Variety**	**Grain yield (t ha** ^**−1**^ **)**	**Grain filling (%)**	**Panicle (m** ^**−2**^ **)**	**Spikelets panicle** ^**−1**^	**Grain weight (mg)**
N1	LYPJ	6.79 ± 0.77 a	86.45 ± 2.32 b	151.11 ± 8.39 a	185.96 ± 7.52 b	23.82 ± 0.53 a
	YLY1	6.81 ± 0.67 a	92.25 ± 0.96 a	146.67 ± 8.81a	180.21 ± 11.29 b	23.67 ± 0.16 a
	YLY2	6.80 ± 0.24 a	84.07 ± 1.09 bc	117.22 ± 15.12 b	260.22 ± 18.52 a	21.67 ± 0.27 bc
	YLY900	7.12 ± 0.09 a	79.74 ± 6.80 c	127.80 ± 5.85 b	273.98 ± 30.23 a	20.95 ± 0.24 c
	S1000	7.59 ± 0.64 a	84.10 ± 1.77 bc	112.22 ± 3.47 b	286.03 ± 44.63 a	21.90 ± 0.58 b
	***Mean***	***7***	***87.4***	***131***	***237***	***22.4***
N2	LYPJ	10.28 ± 1.23 a	79.91 ± 0.55 b	200.33 ± 8.83 abc	218.74 ± 13.55 b	24.70 ± 0.37 a
	YLY1	11.17 ± 0.49 a	89.86 ± 1.52 a	212.22 ± 5.36 ab	195.46 ± 16.31 b	24.80 ± 0.18 a
	YLY2	10.37 ± 0.44 a	75.46 ± 5.61 bc	222.78 ± 29.92 a	233.88 ± 28.20 b	22.51 ± 0.54 b
	YLY900	9.90 ± 0.17 a	71.52 ± 6.36 c	188.88 ± 14.18 bc	287.34 ± 22.98 a	21.66 ± 0.08 c
	S1000	10.52 ± 0.61 a	74.42 ± 6.70 bc	175.00 ± 21.67 c	285.10 ± 17.86 a	22.84 ± 0.24 b
	***Mean***	***10.5***	***81.9***	***200***	***244***	***23.3***
N3	LYPJ	10.08 ± 1.05 b	81.11 ± 1.87 b	203.89 ± 8.55 cd	195.50 ± 23.30 b	24.03 ± 0.27a
	YLY1	9.97 ± 0.78 b	89.49 ± 3.17 a	244.44 ± 8.22 a	165.29 ± 23.55 c	24.86 ± 0.24a
	YLY2	9.68 ± 0.55 b	72.36 ± 0.35 c	191.36 ± 4.55 d	243.46 ± 9.81 a	22.18 ± 0.15b
	YLY900	11.80 ± 0.33 a	73.64 ± 0.54 c	215.59 ± 6.75 bc	230.19 ± 14.41 a	22.07 ± 0.34 b
	S1000	10.33 ± 0.32 b	74.52 ± 5.66 bc	222.78 ± 6.74 b	245.06 ± 18.68 a	22.43 ± 0.72 b
	***Mean***	***10.4***	***78.2***	***216***	***216***	***23.1***
N4	LYPJ	8.96 ± 0.93 a	80.86 ± 3.94 ab	242.22 ± 22.63 ab	166.66 ± 10.30 c	23.28 ± 0.16 a
	YLY1	9.76 ± 0.80 a	83.38 ± 8.64 a	238.89 ± 3.47 ab	181.09 ± 4.30 bc	23.50 ± 1.41 a
	YLY2	9.69 ± 0.33 a	75.04 ± 2.52 b	225.56 ± 12.05 b	232.15 ± 4.63 a	21.73 ± 0.07 b
	YLY900	9.06 ± 0.32 a	75.70 ± 4.04 ab	254.44 ± 17.35 a	236.70 ± 30.23 b	22.29 ± 0.40 ab
	S1000	9.54 ± 0.76 a	73.00 ± 2.23 b	196.11 ± 10.05 c	256.60 ± 4.80 a	22.62 ± 0.13 ab
	***Mean***	***9.4***	***80.6***	***231***	***208***	***22.7***
Analysis of variance						
Variety(V)			**			
N rate(N)			*			
VxN			*			

^†^Within each column, means followed by the same letters were not significantly different according to LSD at P = 0.05. **^,^ *and ns denote significance at the 0.01 and the 0.05 levels and non-significance, respectively, based on analysis of variance.

**Table 2:** Grain yield and yield components of the five super hybrid rice cultivars under the four N treatments in 2016.

**N rate**	**Variety**	**Grain yield (t ha** ^**−1**^ **)**	**Grain filling (%)**	**Panicle (m** ^**-2**^ **)**	**Spikelets panicle** ^**−1**^	**Grain weight (mg)**
N1	LYPJ	7.49 ± 1.08 a	66.04 ± 3.74 b	181.03 ± 19.99 a	194.2 ± 29.00 b	23.20 ± 0.20 ab
	YLY1	8.01 ± 0.42 a	78.70 ± 2.53 a	186.40 ± 22.05 a	173.00 ± 3.40 b	24.07 ± 0.06 a
	YLY2	7.76 ± 0.89 a	81.22 ± 4.32 a	155.83 ± 13.75 ab	244.00 ± 7.90 a	22.63 ± 1.31 bc
	YLY900	7.94 ± 0.91 a	71.50 ± 3.04 b	153.53 ± 9.97 ab	240.70 ± 8.30 a	21.43 ± 0.45 d
	S1000	7.66 ± 0.82 a	69.39 ± 6.41 b	145.13 ± 18.48 b	260.40 ± 9.70 a	21.87 ± 0.61 cd
	***Mean***	***7.7***	***73.4***	***164***	***222***	***22.6***
N2	LYPJ	8.10 ± 1.45 b	67.36 ± 3.23 b	237.57 ± 11.53 ab	180.1 ± 17.8 c	23.37 ± 0.12 b
	YLY1	9.59 ± 0.34 a	77.07 ± 6.45 a	258.30 ± 18.61 a	134.4 ± 0.50 d	24.13 ± 0.25 a
	YLY2	8.97 ± 0.65 ab	72.89 ± 3.89 ab	220.00 ± 17.27 bc	175.6 ± 21.4 c	22.07 ± 0.21 c
	YLY900	10.29 ± 0.87 a	72.99 ± 2.60 ab	165.00 ± 10.54 d	243.3 ± 9.90 b	22.20 ± 0.40 c
	S1000	8.94 ± 0.88 ab	66.91 ± 5.34 b	204.7 ± 18.68 c	290.7 ± 36.4 a	21.73 ± 0.15c
	***Mean***	***9.2***	***71.4***	***217***	***205***	***22.7***
N3	LYPJ	8.21 ± 1.55c	69.98 ± 3.16 b	223.07 ± 22.24ab	192.90 ± 3.90 b	23.43 ± 0.32 b
	YLY1	10.24 ± 0.53 ab	79.06 ± 5.05 a	242.17 ± 29.55 a	181.30 ± 22.90 b	24.30 ± 0.44 a
	YLY2	9.75 ± 0.70 abc	77.16 ± 1.24 a	223.03 ± 16.26 ab	199.80 ± 0.40 b	22.33 ± 0.23 c
	YLY900	11.02 ± 0.64 a	81.97 ± 0.76 a	234.50 ± 19.78 a	232.90 ± 18.50 a	21.53 ± 0.15 d
	S1000	8.80 ± 0.75 bc	65.58 ± 2.34 b	193.27 ± 18.49 b	251.50 ± 15.60 a	21.87 ± 0.25 cd
	***Mean***	***9.6***	***74.8***	***223***	***211***	***22.7***
N4	LYPJ	9.19 ± 0.75 bc	64.78 ± 3.36 b	256.67 ± 17.32 a	196.60 ± 21.40 b	23.53 ± 0.59 a
	YLY1	10.53 ± 0.42 a	79.03 ± 0.84 a	252.07 ± 36.03 a	183.90 ± 31.90 b	24.37 ± 0.31 a
	YLY2	9.80 ± 0.47 ab	72.60 ± 6.84 a	217.70 ± 17.87 a	185.30 ± 13.20 b	22.37 ± 0.35 b
	YLY900	9.20 ± 0.18 bc	76.28 ± 1.44 a	213.90 ± 19.45 a	242.60 ± 11.20 a	21.8 ± 0.40 bc
	S1000	8.21 ± 0.89 c	60.52 ± 4.36 b	222.30 ± 7.97 a	249.60 ± 16.90 a	21.5 ± 0.36 c
	***Mean***	***9.4***	***70.6***	***233***	***212***	***22.7***
Analysis of variance						
Variety(V)			**			
N rate(N)			**			
VxN			ns			

^†^Within each column, means follower by the same letters were not significantly different according to LSD at P = 0.05. **^,^ *and ns denote significance at the 0.01 and the 0.05 levels and non-significance, respectively, based on analysis of variance.

The rates N had significant effects on effective panicles and spikelets per panicle in the two years. High N rate was partially responsible for the large number of effective panicles and spikelets per panicle, while it also resulted in the decrease of grain filling. The number of effective panicles and spikelets per panicles was much higher in YLY900 and S1000 under all N treatments compared with remaining cultivars in the two years.

### Dynamic root morphological traits of five super hybrid rice under four N treatment

**Figure 2:**
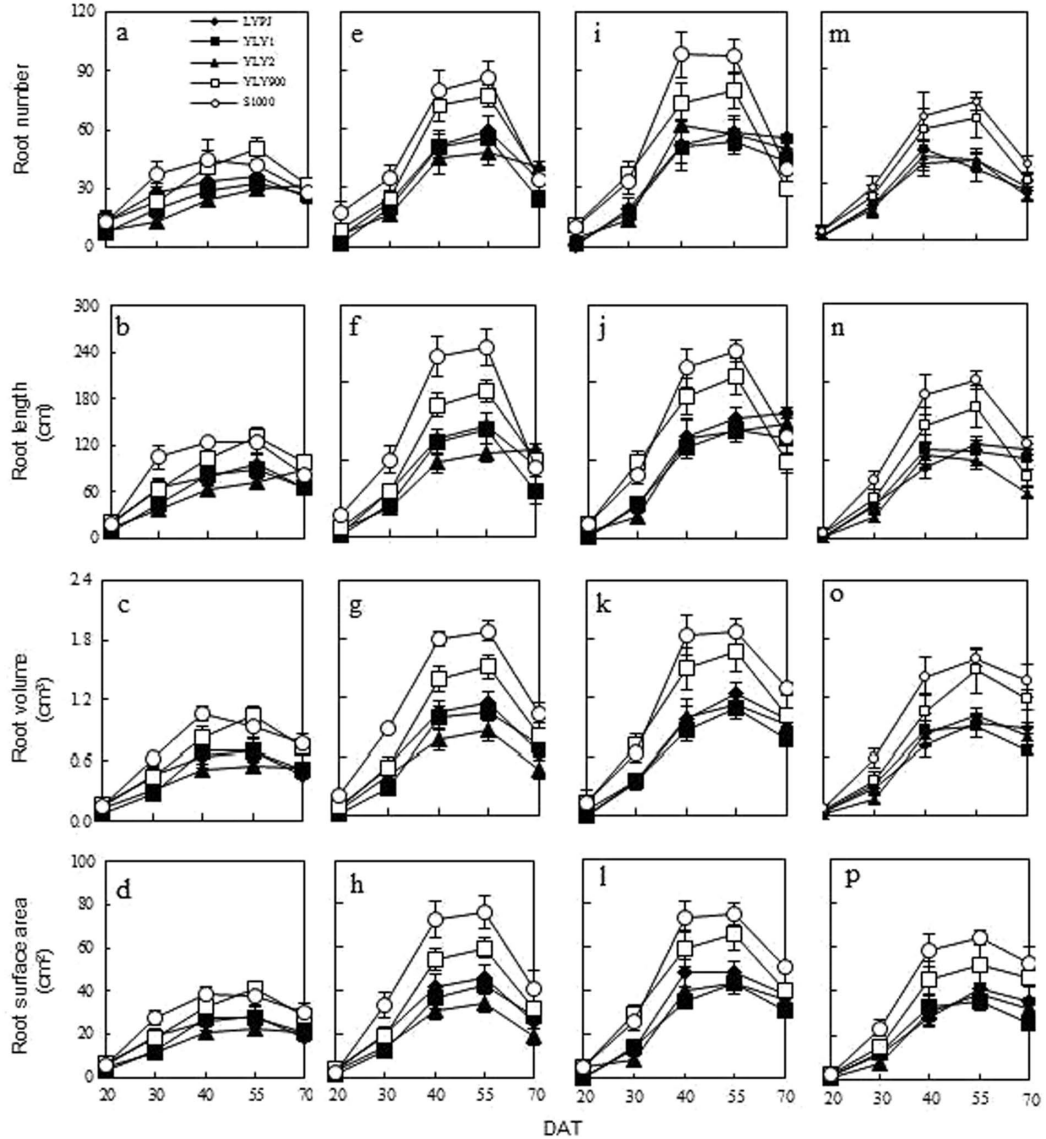
Dynamic root morphological traits of five super hybrid rice cultivars under four N treatments from 20 DAT to 70 DAT in Jingzhou, Hubei Province, China. Data are the means across two years. **N1:** 0 kg ha^−1^ **N2:** 210 kg ha^−1^ **N3:** 300 kg ha^−1^ N4: 390 kg ha^−1^; (**a**), (**e**),(**i**) and (**m**) represent root number; (**b**), (**f**), (**j**) and (**n**) represent root length; (**c**), (**g**), (**k**) and (**o**) represent root volume; (**d)**,(**h)**,(**l)** and (**p**) represent root surface area; (**a**–**d**), (**e**–**h**), (**i**–**l**) and (**m-p**) mean different root traits under N1, N2, N3 and N4, respectively. Error bars above mean indicate standard error (n = 8).

The rate of N had significant effects on TRN of the five super hybrid rice cultivars (Fig. 2). Compared with N1, the mean TRN of these cultivars significantly increased under nitrogen treatments, showing an increase of 39.87% (N2), 54.06% (N3) and 15.99% (N4), respectively. The TRN of these cultivars reached maximum at 55 DAT, and the TRN of YLY900 and S1000 was significantly higher than other super hybrid rice cultivars across various nitrogen treatments.

Similar to TRN, the TRL of the five super hybrid rice cultivars was also significantly affected by nitrogen treatments (Fig. 2). The mean TRL of these cultivars under N3 increased by 43.42%, which was higher than N2 (38.30%) and N4 (22.11%). YLY900 and S1000 showed a significantly greater decrease in the TRL than other cultivars from 55 to 70 days after transplanting across various nitrogen treatments.

The TRV of the five super hybrid rice cultivars significantly increased with the addition of nitrogen (Fig. 2). The mean TRV of these cultivars under N3 was 4.2 cm^3^, which was higher than N2 (3.9 cm^3^) and N4 (3.7 cm^3^). The TRV of YLY900 and S1000 was much higher than other cultivars across different nitrogen treatments, but showed a greater decrease rate than other cultivars from 55 to 70 days after transplanting.

The dynamic changes of TRSA in the five cultivars under different nitrogen treatments showed the same trend as TRV (Fig. 2). The response of the TRSA of these cultivars to the nitrogen treatment was significant in YLY900 and S1000, while other cultivars showed a slight increase. The TRSA of YLY900 reached the highest at 65.75 cm^2^ under N3 at 55 DAT under N3, but S1000 reached the highest at 75.83 cm^2^ under N2 treatment.

### Root spatial distribution of five super hybrid rice under four N treatments

**Figure 3:**
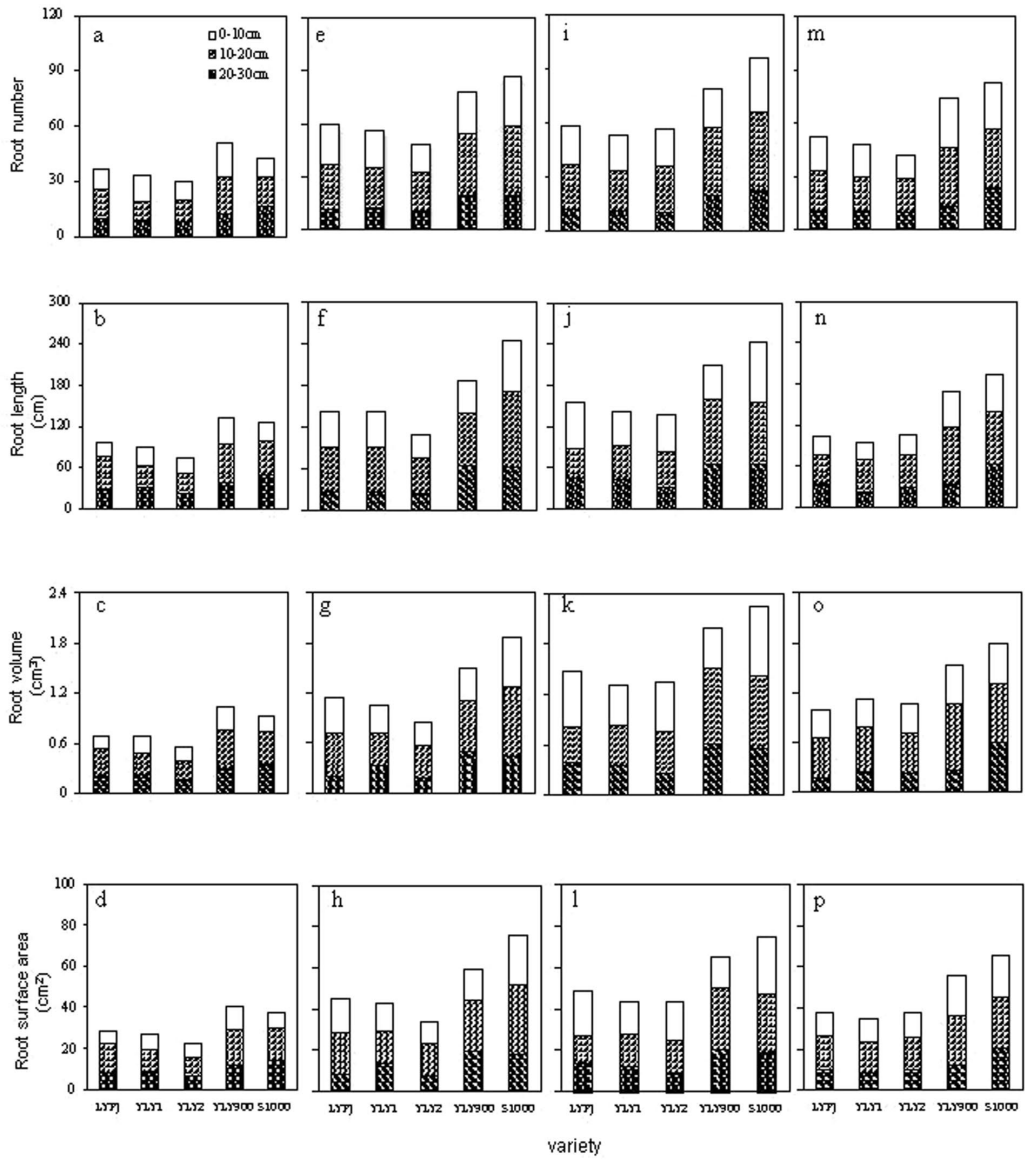
Root spatial distribution (0–30 cm soil layer) of five super hybrid rice cultivars under four N treatments at 55 DAT in Jingzhou, Hubei Province, China. Data are the means across two years. **N1:** 0 kg ha^−1^ **N2:** 210 kg ha^−1^ **N3:** 300 kg ha^−1^ N4:390 kg ha^−1^; (**a**), (**e**),(**i**) and (**m**) represent root number; (**b**), (**f**), (**j**) and (**n**) represent root length; (**c**), (**g**), (**k**) and (**o**) represent root volume; (**d**), (**h**), (**l**) and (**p**) represent root surface area; (**a**–**d**), (**e**-**h**), (**i**–**l)** and (**m–p**) mean different root traits under N1, N2, N3 and N4, respectively.

The TRN of these cultivars on different soil layers increased significantly among various nitrogen treatments, especially at the 10–20 cm soil layer where the number of roots was much higher than the other soil layers (Fig. 3). The mean TRN of all cultivars under N3 was the highest (69.1), which was 5.66% and 15.46% higher than those of N2 and N4, respectively. The proportion of TRN at 10–20 cm in YLY900 and S1000 was 47.48% and 45.88%, respectively, under N3 treatment, much higher than other cultivars.

Nitrogen treatments significantly affected the TRL of super hybrid rice cultivars in different soil layers (Fig. 3). The TRL of the five super hybrid rice cultivars increased significantly with the addition of nitrogen. The mean TRL of these cultivars under N3 was the highest (176.2 cm), 5.88% and 33.82% higher than N2 and N4, respectively. The proportion of root length at 10–20 cm in YLY900 and S1000 was 45.76% and 37.37%, respectively, under N2 treatment, being much higher than other cultivars.

Nitrogen treatments had a significant effect on the TRSA of five super hybrid rice cultivars (Fig. 3). The mean TRSA of these cultivars under N3 was the highest (60.5 cm^2^). The proportions of TRSA at 10–20 cm in YLY900 (45.73%) and S1000 (37.93%) under N2 treatment were much higher than other cultivars.

The TRV on different soil layers of the five super hybrid cultivars was also significantly affected by nitrogen treatments (Fig. 3). The mean TRV of these cultivars under N3 was the highest. The TRN of YLY900 and S1000 was significantly higher than other cultivars. Similar to TRSA, the proportion of TRV at 10–20 cm in these cultivars under N4 was higher than that under N2.

### Relationship between spatial distribution of root system and yield components

The TRL, TRN, TRSA and TRV were positively correlated with grain yield, spikelets per panicle and panicle per m^2^, but these root characteristics were negatively correlated with grain filling (Table 3). The TRL and TRN at 10–20 cm soil layer were significantly correlated with grain yield (*P* < 0.01), with correlation coefficients at 0.56 and 0.68, respectively, and the TRSA and TRV at 0–10 cm soil layer were significantly correlated with grain yield (*P* < 0.01).

**Table 3:** Correlation coefficients(r) between root morphological characteristics and yield components.

Root traits	Soil Layer	Grain filling	Panicle	Spikelets panicle^−1^	Grain weight	Grain yield
	L_0–10 cm_	−0.58**	0.47*	0.19	0.04	0.65**
TRL	L_10–20 cm_	−0.72**	0.36	0.42	−0.29	0.56**
	L_20–30 cm_	−0.50*	0.16	0.41	−0.20	0.45*
	L_0–10 cm_	−0.58**	0.56**	0.13	0.05	0.65*
TRN	L_10–20 cm_	−0.65**	0.42	0.32*	−0.12	0.68**
	L_20–30 cm_	−0.65**	0.17	0.47*	−0.27	0.48*
	L_0–10 cm_	−0.53*	0.45*	0.14	−0.05	0.63**
TRSA	L_10–20 cm_	−0.65**	0.33	0.38	−0.22	0.55*
	L_20–30 cm_	−0.48*	0.16	0.39	−0.21	0.47*
	L_0–10 cm_	−0.61**	0.48*	0.17	−0.08	0.70**
TRV	L_10–20 cm_	−0.62**	0.41	0.36	−0.19	0.67**
	L_20–30 cm_	−0.54*	0.29	0.35	−0.27	0.49

^†^_T_he values refer to the correlation coefficients. **and *denote significance at the 0.01 and the 0.05 levels, respectively.

## Discussion

In our study, the representative super hybrid rice cultivars showed improved yield potential for a larger sink size under different nitrogen treatments, although the grain yield of these cultivars among various nitrogen treatments was lower than target yield. This large yield gap could be attributed to several reasons. Firstly, it has been reported that favorable environmental conditions are essential for achieving high yield potential of super hybrid rice^27^. Many of those high yield records were mainly achieved in only a few high-yielding environments which have cool air temperature and high accumulative solar radiation during grain filling period^28,29^. The two key environmental factors mentioned above in our experimental sites in Jingzhou city are significantly different from those of specific ecosystem (data not shown). Secondly, the abnormal weather has occurred frequently in the past few years in Jingzhou during the grain filling period of rice. Mean daily maximum and minimum temperatures during the anthesis reached a new high in 2016. Plus, Jiang *et al*., reported that higher fertility soil was partially responsible for the lager yield advantage of hybrid rice, and soil indigenous N was more important than fertilizer N^11^. However, soil characteristics (soil organic matter, total N and available K contents) at the current experimental site were also lower than those specific ecosystem. These unfavorable climates and infertility soils were partially responsible for the significant yield difference.

The present results showed that there were positively correlated between grain yield and N rates within a certain range (0–210 kg ha^-1^), whereas the grain yield showed less increase or even decreased once the N rate was over 210 kg ha^-1^. It was reported that over application of N fertilizer may actually decrease grain yield by increasing susceptibility to lodging^30^. However, lodging is not the critical factor which led to the lower yield under high nitrogen levels in the current study, because no lodging occurred in the field in both years. This difference was possibly due to that most newly-released cultivars were selected under high nitrogen conditions thus have better lodging resistance. There are several reasons that higher N application rates resulted in a lower yield in this study. Over-application of N fertilizer could lead to over-luxuriant growth of rice leaves^31^ thus older leaves are more or less shaded by upper young leaves, which can be detrimental to photosynthesis efficiency. Consequently, the progress of photosynthate transferred directly to the grain and photosynthate redistributed from reserve pools in vegetative tissues was inhibited. Another reason is that high rates of N fertilizer promote luxuriant vegetative growth with dense foliar canopies. This type of canopy structure provides a favorable environment for pest development^32^. In the constant two years, both sheath blight and rice planthopper incidence increased with increasing N level, especially at the highest N level plots in the field.

Most published researches related to root morphology focused on root oxidation activity and root morphological characteristics^20,33^. As such, little information was available on the dynamic characteristics of roots based on real field conditions. Likewise, many scientific methods aiming at root research mainly concentrated on invasive sampling in the paddy fields, water or sand culture method for growing rice plants. These approaches have their weaknesses since they do not necessarily reproduce field conditions or possible confounding effects of other soil factors apart from texture. In our study, dynamic characteristics of roots in super hybrid rice from 20 days after transplanting to anthesis were studied with Minirhizotron techniques. Our results showed the root growth of these super hybrid rice cultivars tended to decrease when subjected to high nitrogen treatment (N4). Similar result was also observed by previous research which reported that moderate nitrogen treatment could stimulate root growth more strongly in rice than high nitrogen treatment^25,27^. In our study, there were great differences among various super hybrid rice cultivars in terms of root growth rate, nitrogen requirement for root growth and nitrogen absorbing ability of roots. These findings suggest that varietal differences were partially responsible for different responses to nitrogen treatments among these cultivars. Although nitrogen topdressing at critical growth stages could have significant effects on root growth, fertilizer requirements of cultivars should be taken into consideration in determining fertilizer rate in the agricultural production sector, thereby making it possible to achieve high yield potential of super hybrid rice by regulating rice root growth with suitable nitrogen managements.

Several studies have been conducted on the effects of different nitrogen treatments on root morphological characteristics in super hybrid rice but only few described the dynamic changes of root distribution in different soil layers in super hybrid rice^20,27^. Previous studies also demonstrated that rice roots are densely distributed over the plough layer (0–20 cm) and roots in topsoil (0–10 cm) account for over 80% of the whole root numbers^34^. Our results, however, showed that the four root characteristics at 10–20 cm soil layer significantly increased under various nitrogen treatments though root distribution was significantly different in soil layers. The root numbers on 0–10 cm soil layer merely accounted for 25%-30% of the whole root numbers, but over 50% on the 10–20 cm soil layer. This difference could be explained by the difference in the cultivars tested. Compared with inbred cultivars, super-high-yielding rice variety displays the following morphological features such as greater panicle size and higher shoot biomass^15^. When the plough layer is shallow, roots are densely distributed in the topsoil; Therefore, root lodging occurs frequently in this condition, which can cause damage to grain yield of rice^35^. Super hybrid rice cultivars are more likely to lodge because of insufficient anchorage and drooping panicle. Based on these special characteristics, research was aimed at the improvement of root numbers and root surface areas at deep soil layers during the evolution of super hybrid rice breeding, which can improve lodging resistance and grain filling in super hybrid rice. This hypothesis was confirmed in our study, and an improved root system was found in YLY900 and S1000 showing greater TRN, TRL, TRSA and TRV at early and mid-growth stages than the other three cultivars. Our results also confirmed earlier reports that the number of roots at deep soil layer was positively correlated with grain yield in rice^36^. In this study, the mean grain yield under N2 in the two years was higher than other nitrogen treatments. More importantly, the mean proportion of TRN and TRL in five super hybrid rice cultivars at 10–20 cm soil layer was at 43.8% and 44.2% under the same condition, respectively, which were also higher than other treatments. Significantly positive correlations were found between grain yield and the TRN, TRL and TRSA at 10–20 cm soil layer (*P* < 0.01). These findings suggest that the roots at 10–20 cm soil layer may play an important role in realizing the high yield potential of super hybrid rice. A relatively slight reduction in the grain yield and poor root characteristics of these super hybrid rice cultivars was noticed under N4. Further research is needed to explore the mechanism involved in poor root morphological characteristics and yield fluctuation caused by excessive nitrogen treatment and to improve the yield performance in super hybrid rice cultivars in the future.

## Material and Methods

Field experiments were conducted at the experimental farm of Yangtze University (112°31′E, 30°21′N) in Jingzhou City, Hubei Province, China, in 2015 and 2016. The soil of the experimental site was a calcareous alluvial with the following properties: pH: 6.8; organic matter: 18.5 g/kg; alkali-hydrolyzale N:110.5 mg/kg; available P: 25.0 mg/kg; available K: 105.5 mg/kg; The soil test was based on the samples taken from the upper 20 cm of the soil.

Treatments were arranged in a split-plot designed with N treatments as the main plots and cultivars as subplots. The experiment was replicated three times, and the subplot size was 30 m^2^. The four N treatments were 0 kg ha^−1^ (N1), 210 kg ha^−1^ (N2), 300 kg ha^−1^ (N3) and 390 kg ha^−1^ (N4). For the N2 treatment, 105, 42 and 63 kg N/ha were applied as baseline one day before transplanting, at early tillering (7 days after transplanting), and panicle initiation (the first appearance of a differentiated apex), respectively. For the N3 treatment, 150, 60 and 90 kg N/ha were applied at baseline, early tillering, and panicle initiation, respectively. For the N4 treatment, 195, 78 and 117 kg N/ha were applied at baseline, early tillering, and panicle initiation, respectively.

Five super hybrid rice cultivars were used in the experiments. These cultivars have been widely grown by rice farmers in China (http://www.ricedata.cn/). Detailed information about these varieties is given in Table 4. Pre-germinated seeds of Yliangyou-900 and Super−1000 were sown in a seedbed at a rate of 25 g m^-2^ on 30 April and the other seeds were sown on 5 May 2015. In 2016, Yliangyou-900 and Super−1000 were sown with the same rate on 4 May and the other seeds were sown on 9 May. Seedlings were transplanted at a hill spacing of 20 cm × 30 cm with two seedlings per hill. Phosphorus (58, 83 and 108 kg P /ha) was applied and incorporated in all subplots one day before transplanting under N2, N3 and N4, respectively. Potassium (12, 17 and 22 kg K /ha) was split equally at basal and panicle initiation under N2, N3 and N4, respectively. Crop management followed the standard cultural practices. The experimental field was kept flooded from transplanting until 10 days before maturity. Insects were intensively controlled by chemicals to avoid biomass and yield loss.

**Table 4:** Information about selected cultivars in our experiment in 2015 and 2016.

Variety	Type	Year of release	Female parent	Male parent
LYPJ	Intermediate†	1999	Pei’ai64S	9311
YLY1	Indica	2006	Y58S	9311
YLY2	Indica	2006	Y58S	Yuanhui2
YLY900	Indica	2015	Y58S	R900
S1000	Indica	unreleased	Guangxiang24S	R900

^†^The intermediate indicates the genotype between indica and japonica.

Twelve hills were sampled diagonally from a 5-m^2^ harvest area in each subplot at maturity. For all the sampling procedures, the three external lines were excluded to avoid border effects. Plants were hand threshed after the panicles were counted. Filled grains were separated from unfilled grains by submerging them in tap water. Three subsamples of 30 g filled grains and all unfilled grains were taken to count the numbers of spikelets. The filled and unfilled grains were determined after oven drying at 70 °C to constant weight. Spikelets per panicle and grain filling percentage (100 x filled grain number/total grain numbers) were calculated. Grain yield was determined from the same 5-m^2^ area and adjusted to a moisture content of 0.14 g H_2_O g^−1^ fresh weigh.

Root measurements were made with the CI-600 root growth monitoring system (CID Bio-Science-CI-600, Camas, WA, USA) fitted with a scanner head for collecting images, a laptop computer and 1 m standard clear soil tubes (50.8 mm internal diameter) with end caps. In 2015 and 2016, two plastic tubes were installed on a permanent basis to harvest at the centre of each plot two days after transplanting. Each tube was 10 cm apart away from rice plant, and the distance between the two tubes was 1.5 m in each plot. An auger of the same external diameter as the tube was used to facilitate close tube soil contact. In turn, the scanner was inserted into each tube to the depth of 60 cm. Images were captured at three depths with the aid of an automatic indexing handle, equivalent (given the angle of the tube at 30^◦^ off vertical) to 0–10, 10–20 and 20–30 cm. Each scan provides a nearly 360^◦^ image (21.59 × 19.56 cm) with a resolution of 200 dpi. Images were captured at 20, 30, 40, 55 and 70 days after transplanting. Total root length (TRL, cm), total number of roots (TRN), total root surface area (TRSA, cm^2^) and total root volume (TRV, cm^3^) per sample and tube segment for each plot were calculated from these images processed by WinRhizotron® software (Regent Instruments Inc., Canada).

Daily minimum and maximum temperature data were obtained from the local meteorological bureaus.

### Statistical analysis

Data were analyzed following analysis of Variance (Statistix 8, Analytical Software, Tallahassee, FL, USA), and means of cultivars were compared based on the least significant difference test (LSD) at the 0.05 probability level.
